# Considerations for the design of antibody drug conjugates (ADCs) for clinical development: lessons learned

**DOI:** 10.1186/s13045-023-01519-0

**Published:** 2023-12-12

**Authors:** Alfonso López de Sá, Cristina Díaz-Tejeiro, Elisa Poyatos-Racionero, Cristina Nieto-Jiménez, Lucía Paniagua-Herranz, Adrián Sanvicente, Emiliano Calvo, Pedro Pérez-Segura, Víctor Moreno, Francisco Moris, Alberto Ocana

**Affiliations:** 1 Medical Oncology Department, Hospital Clínico Universitario San Carlos, Instituto de Investigación Sanitaria San Carlos (IdISSC), and CIBERONC, Madrid, Spain; 2 Experimental Therapeutics in Cancer Unit, Instituto de Investigación Sanitaria San Carlos (IdISSC), Madrid, Spain; 3 Cancerappy S.L., 48950 Erandio, Biscay Spain; 4 Facultad de Ciencias Químicas, Universidad Complutense de Madrid, 28040 Madrid, Spain; 5 START Madrid-HM Centro Integral Oncológico Clara Campal (CIOCC), Early Phase Program, HM Sanchinarro University Hospital, Madrid, Spain; 6 START Madrid-Fundación Jiménez Díaz (FJD) Early Phase Program, Fundación Jiménez Díaz Hospital, Madrid, Spain

## Abstract

Antibody–drug conjugates (ADCs) have emerged as a novel therapeutic strategy that has successfully reached patient treatment in different clinical scenarios. ADCs are formed by an antibody against a specific tumor-associated antigen (TAA), a cytotoxic payload, and a chemical linker that binds both. To this regard, most efforts have been focused on target identification, antibody design and linker optimization, but other relevant aspects for clinical development have not received the necessary attention. In this article using data from approved ADCs, we evaluated all characteristics of these agents, including payload physicochemical properties, in vitro potency, drug antibody ratio (DAR), exposure–response relationships, and clinical development strategies. We suggest that compounds with best options for clinical development include those with optimal payload physicochemical properties and cleavable linkers that would lead to a bystander effect. These modalities can facilitate the development of ADCs in indications with low expression of the TAA. Early clinical development strategies including changes in the schedule of administration with more frequent doses are also discussed in the context of an efficient strategy. In conclusion, we highlight relevant aspects that are needed for the optimal development of ADCs in cancer, proposing options for improvement.

**Supplementary Information:**

The online version contains supplementary material available at 10.1186/s13045-023-01519-0.

## Introduction

The use of monoclonal antibodies (mAbs) as a therapeutic modality has gained momentum as several of these agents have demonstrated to improve patient survival in recent years. The first antibodies designed for the treatment of cancer were developed against oncogenic proteins expressed at the surface of the cell membrane. Examples of them include, among others, trastuzumab (Tz), against HER2 or cetuximab, against EGFR [1]. Given the role of these protein kinases in cancer, the inhibition of their kinase activity with small molecules also produced antitumoral activity and was exploited therapeutically [2, 3]. The mechanism of action of these antibodies was mainly related to the reduction of the amount of the target on the cellular membrane secondary to an endocytotic process [4–6]. Indeed, antibodies designed specifically against the interacting-ligand domain, as was the case for pertuzumab, did not demonstrate enhanced activity, compared with antibodies against other extracellular regions, or compared with biparatopic antibodies with enhanced activity [7]. This suggested the importance for an efficient internalization and endocytosis of the receptor as the principal mechanisms of action.

Much more recently, antibodies have been used to guide cytotoxic compounds, or payloads, that were attached to the antibody by a chemical linker [8]. This family of agents has been termed antibody–drug conjugates (ADCs), and at this moment more than eleven agents have demonstrated meaningful clinical activity and therefore have received regulatory approval [5]. In addition, more than one hundred are currently in clinical development in the USA, Asia and Europe [9].

ADCs are three-component structures, whose three different elements must function correctly to deliver the full potential of their mechanism of action. The selectivity and specificity of the antibody is crucial, as it is expected to act only on the tumor-associated antigen (TAA). In this context, the TAA would preferentially be expressed in tumoral cells in a homogenous manner [10]. The linker should efficiently deliver the payload (by releasing it or not), and finally the drug payload should have proper physicochemical and antitumoral characteristics [6, 11]. For this last component, most of them have historically been chemotherapy drugs, either DNA-damaging agents, or agents that induce cell cycle arrest, and mainly act at very low concentrations due to its intrinsic toxicity [11]. In this article, using data from approved ADCs, we have performed a detailed analysis of the characteristics of each component, to understand current limitations and suggest future modifications that could improve clinical development. The methodology and data used for the analysis provided in this article are available in as Additional file 1.

### Tumor-associated antigen (TAA) expression of approved ADCs across different indications

**Fig. 1:**
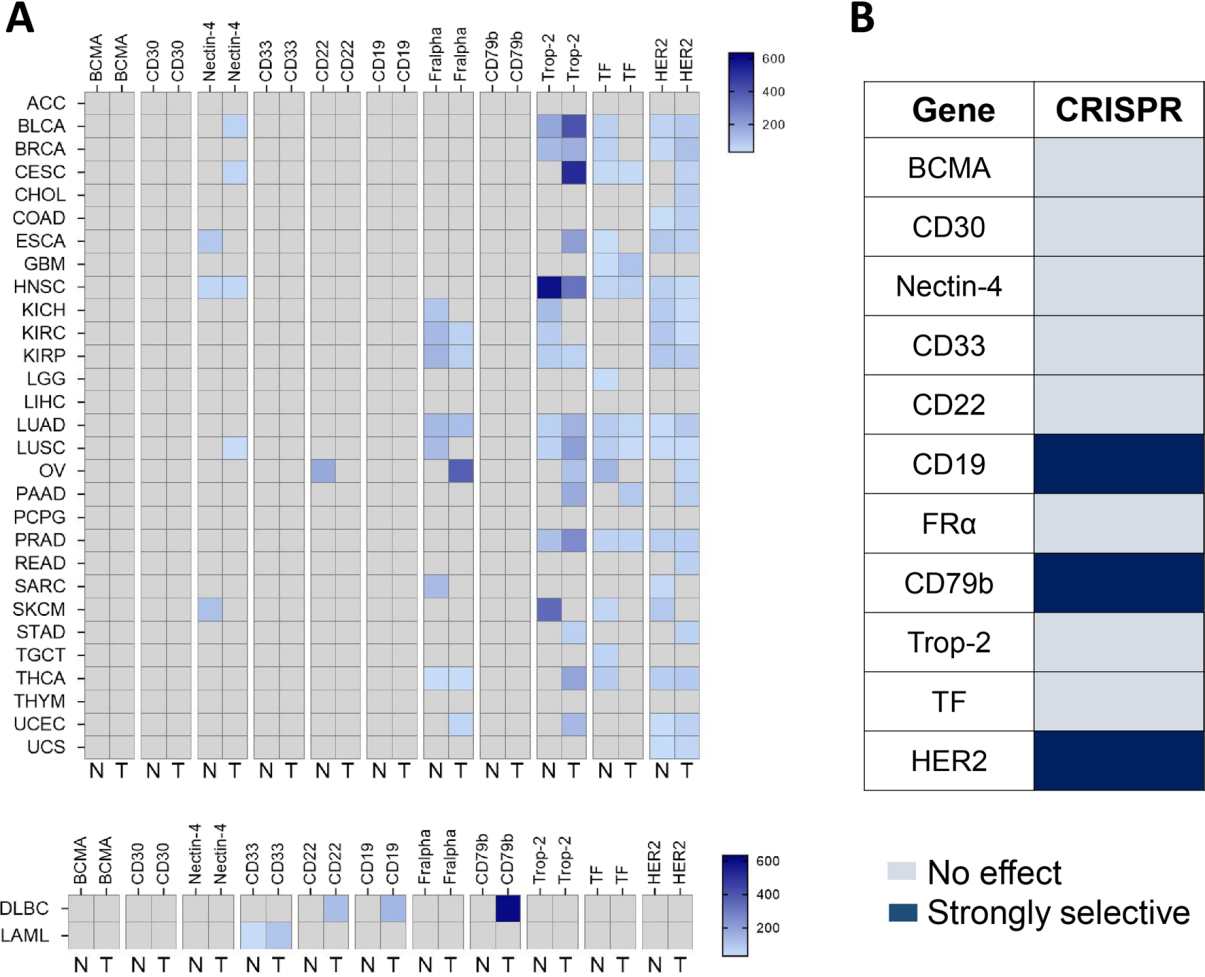
Tumor-associated antigen (TAA) expression of approved ADCs across different indications; **a** expression of normal and tumoral tissue expressed in transcript per million (TPM) (GEPIA2), **b** effect on cell viability by CRISPR silencing in different cell lines (DepMap)

Selective expression of the TAA in tumoral cells is key to avoid on-target off-tumor toxicities. In this context, using transcriptomic data, we mapped the expression of all the TAA for which ADCs has been approved. We first evaluated the expression of each target at a transcriptomic level in normal tissue versus tumors. As can be seen in Fig. 1a those targets belonging to hematological malignancies were mainly expressed in tumors and not in other tissues, including CD33, CD19 and CD22, among others. By contrast, for solid malignancies, the expression of TAA in normal tissue was more evident including targets like FR-alpha, Trop2, Nectin-4 or TF, among others (Fig. 1a). As TAA can also play an oncogenic role, we evaluated which of the evaluated targets was considered as a common essential gene. As can be seen in Fig. 1b, CD19, CD79b and HER2 were considered as strongly selective genes in several cell lines.

### Type of linker

All approved ADCs except two, belantamab mafodotin (Blenrep) and trastuzumab emtansine (Kadcyla), have used linkers with cleavable structures. Among all the cleavable linkers, most of them were associated with enzymatic activity and only two were pH dependent (Table 1).

**Table 1:** Antibody drug conjugate (ADC) characteristics: linker class, payload and payload mechanism of action, drug antibody ratio (DAR), potency of the payload and of the ADC (IC50, nM) expressed as a range in sensitive and non-sensitive cell lines and ratio of IC50 ADC/payload in sensitive cells only

ADC	Linker	Payload	Type of payload	DAR	IC50 range payload, nM	IC50 range ADC, nM	IC50 ration ADC/payload-sensitive cell lines
Belantamab mafodotin (Bienrep)	Non-cleavable linkers	MMAF, Auristatin	Tubulin polymerization promoters	4	100–200	–	
Brentuximab vedotin (Adcetris)	Cathepsin B-sensitive linker	MMAE, Auristatin	Tubulin polymerization promoters	4	0.07–3.1	0.003 − 0.125	0.042 (24 × more potent)
Enfortumab vedotin (Padcev)	Cathepsin B-sensitive linker	MMAE, Auristatin	Tubulin polymerization promoters	3.8	0.07–3.1	0.008–0.28	0.11 (9 × more potent)
Gemtuzumab ozogamicin (Mylotarg)	Hydrazone linker/pH-sensitive linker	Ozogamicin, Calicheamicin	DNA double-strand breaking	2–3	0.01–0.06	0.003—0.084	0.3 (3 × more potent)
Inotuzumab ozogamicin (Besponsa)	Disulfide linker/glutathione-sensitive linker	Ozogamicin, Calicheamicin	DNA double-strand breaking	6	0.01–0.06	0.009–0.43	0.9 (as potent as the payload)
Loncastuximab tesirine (Zynlonta)	Cathepsin B-sensitive linker	SG3199, PBD dimer	DNA crosslinking	2–3	0.15–1	0.002—0.0036	0.013 (77 × more potent)
Mirvetuximab soravtansine (Elahere)	Disulfide linker/glutathione sensitive linker	DM4, Maytansinoid	Tubulin polimerization blockers	3.5	0.03–0.06	0.5—24	16 × less potent
Polatuzumab vedotin (Polivy)	Cathepsin B-sensitive linker	MMAE, Auristatin	Tubulin polymerization promoters	3.5	0.07–3.1	0.07	1 (as potent as the payload)
Sacituzumab govitecan (Trodelvy)	Hydrazone linker/pH-sensitive linker	SN-38, Topoisomerase inhibitor	DNA intercalation	7.6	13–700	0.2- 0.3	0.015 (66 × more potent)
Tisotumab vedotin (Tivdak)	Cathepsin B-sensitive linker	MMAE, Auristatin	Tubulin polymerization promoters	4	0.07–3.1	0.01–3.8	0.14 (7 × more potent)
Trastuzumab Deruxtecan (Enhertu)	Cathepsin B-sensitive linker	DXd, Topoisomerase inhibitor	DNA intercalation	8	1.7–9.0	0.04—0.16	0.023 (43 × more potent)
Trastuzumab Emtansine (Kadcyla)	Non-cleavable linker	DMl, Maytansinoid	Tubulin polimerization blockers	3.5	0.79–7.2	0.2—29	0.25 (4 × more potent)

There are three types of cleavable linkers. The hydrazone-based linkers experience hydrolysis when exposed to acidic pH, a circumstance that typically occurs when the ADC is transported through endosomes or lysosomes with a pH of 5–6 and 4.8, respectively [12]. The Cathepsin B-sensitive linker is a protease-based linker that is active in the lysosomes [13]. It recognizes certain sequences that can be used as ADC linkers, so the cleavage takes place once the ADC has been internalized inside de lysosome [14]. Finally, disulfide linkers are sensitive to reductive cleavage by glutathione. Indeed, glutathione and other reducing molecules have higher concentrations in the cytoplasm than in the extracellular domain [15]. On the other hand, non-cleavable linkers allow the scission of the linker-payload from the mAb, through a direct degradation of the protein. If this degradation is not completed successfully, some parts of the mAb can remain associated with the linker and payload after the release, impairing the diffusion through the lysosomal and cytoplasmic membranes [16].

### Payload characteristics

Cytotoxic-approved payloads are mainly DNA-damaging agents and tubulin inhibitors. DNA-damaging agents include those that act inducing double-strand breaks like the calicheamicin derivative ozogamicin or the DNA intercalating/crosslinking compounds: SN-38 and DXd (Topoisomerase I inhibitors), or tesirine (Pyrrolobenzodiazepines (PBD) [17]. Tubulin inhibitors include tubulin polymerization promoters such as auristatin derivatives monomethyl auristatin E and F (MMAE and MMAF) and tubulin polymerization blockers like the maytansinoid derivatives, emtansine (DM1) and DM4 [8, 18] (Table 1).

Regarding the payload activity, Calicheamicin (10–60 pM), the maytansinoid DM4 (30–60 pM) and the auristatin MMAE (70 pM–3.1 nM), all of them showed pM potency in sensitive cell lines. Next, several others fall in the subnanomolar or low nanomolar range, as is the case for the maytansinoid DM1 (0.79–7.2 nM), the topoisomerase inhibitor deruxtecan (1.7–9.0 nM) and the PBD dimer SG3199 (0.15–1 nM). There are two payloads whose activity for cell lines is in the submicromolar range, like the auristatin MMAF (100–200 nM) and the topoisomerase inhibitor SN-38 (13–700 nM), which is the active metabolite of the clinically used drug irinotecan.

The potency of the ADC in vitro against panels of cell lines does not necessarily correlate to that of the free payloads. In sensitive cell lines, all ADC potency data were reported below nM levels, many in the single pM potency range (2–9 pM): gemtuzumab ozogamicin (Mylotarg), inotuzumab ozogamicin (Besponsa), Brentuximab vedotin (Adcetris), Enfortumab vedotin (Padcev) and Loncastuximab tesirine (Zynlonta). Except the calicheamicin-based ADCs, for the other three, it represented an improvement in IC50 of about one order of magnitude (almost two orders in the case of Loncastuximab tesirine (Zynlonta)). The next range in potency was represented by three ADCs with double-digit pM activity (10–70 pM): Tisotumab vedotin (Tivdak), trastuzumab deruxtecan (Enhertu) and polatuzumab vedotin (Polivy). In this group the ratio of improvement varies, Polivy had the same IC50 as the payload, Tivdak was about 7 times more potent than the payload and trastuzumab deruxtecan (Enhertu) showed a significant improvement of 40 times over the payload.

The least active ADCs as judged by their in vitro activity were sacituzumab govitecan (Trodelvy) and Tz-emtansine (Kadcyla) both at 200 nM and, finally, mirvetuximab soravtansine (Elahere), at 500 nM. In all cases the improvement in the IC50 does not reach one order of magnitude, and indeed for mirvetuximab soravtansine (Elahere) the most recently approved ADC, the IC50 of the ADC is higher than that of the payload [19].

The activity of an ADC can also depend on other characteristics like the drug/antibody ratio (DAR). As can be seen in Table 1, this can range between 2 and 8 [20]. The conjugation connects accessible lysine or cysteine residues with the linker. However, since lysine residues provide a limited number of linking sites and a particular reactivity, cysteine-based conjugation is preferable, to provide a more controlled DAR [2–8] and stability [21]. It must be remarked that, although a high DAR is related to a high ADC potency, the best DAR is yet to be established [22].

### Physicochemical characteristics of ADC-approved payloads

For decades, the impact that certain physicochemical properties such as lipophilicity have on the biological activity of a drug has been known [23]. These principles can also be applied to the payload of the ADCs, since at some point in their mechanism of action that payload will be released. In this context, the physicochemical characteristics of the different payloads were evaluated to determine their possible impact on the efficiency of each ADC.

As can be seen in Table 2 all compounds except trastuzumab deruxtecan (Enhertu) and sacituzumab govitecan (Trodelvy) violated one or several parameters of the Lipinski rules, mainly due to the high molecular weight of the payloads, and their high number of hydrogen bond acceptors. All the other payloads had at least two violations, and two ADCs based on calicheamicin (gemtuzumab ozogamicin (Mylotarg) and inotuzumab ozogamicin (Besponsa), showed up to three violations of the rules, since they also exceed the number of hydrogen bond donors.

**Table 2:** Values of the different physicochemical parameters comparing the Lipinski rule, in conjunction with the estimation of the lipophilicity and solubility of the different payloads linked to the ADCs

ADC	Payload	Linker	DAR	Date FDA	Lipinski				Solubility	Lipophilicity		AB-MPS
					MW < 500	MLOGP ≤ 4.15	N or O < 10	NH or OH ≤ 5	Log S average	Log P average ≤ 5	cLog D (ChEMBL)	
Belantamab mafodotin*	MMAF/Auristatin	No cleavable	4	2020	1046.32	− 2.80	14	5	− 6.25	2.25	–	–
Brentuximab vedotin	MMAF/Auristatin	Cleavable enz	4	2011	717.98	0.69	8	4	− 6.20	3.47	2.01	30.99
Enfortumab vedotin	MMAF/Auristatin	Cleavable enz	3.8	2019	717.98	0.69	8	4	− 6.20	3.47	2.01	30.99
Gemtuzumab ozogamicin	Ozogamicin/Calicheamicin	Cleavable pH	2–3	2000 and 2017	1368.35	− 2.06	23	8	− 7.88	2.89	3.11	31.11
Inotuzumab ozogamicin	Ozogamicin/Calicheamicin	Cleavable pH	6	2017	1368.35	− 2.06	23	8	− 7.88	2.89	3.11	31.11
Loncastuximab tesirine	SG3199/PBD dimer	Cleavable enz	2.3	2021	725.79	2.02	9	**1**	− 7.84	4.55	4.1	36.1
Mirvetuximab soravtansine	Maytansinoid DM4	Cleavable	3.5	2022	780.37	2.21	10	2	− 6.49	3.51	4.47	17.47
Polatuzumab vedotin	MMAE/Auristatin	Cleavable enz	3.5	2019	717.98	0.69	8	4	− 6.20	3.47	2.01	30.99
Sacituzumab govitecan	SN-38	Cleavable pH	7.6	2020	392.40	1.55	6	2	− 4.63	2.46	1.87	19.13
Tisotumab vedotin	MMAE/Auristatin	Cleavable enz	4	2021	717.98	0.69	8	4	− 6.20	3.47	2.01	30.99
Trastuzumab deruxtecan	DXd	Cleavable enz	8	2019	493.48	1.26	8	3	− 4.21	1.98	0.51	22.49
Trastuzumab emtansine*	DM1/Auristatin	No cleavable	3.5	2013	1103.71	− 2.07	16	5	− 6.17	1.82	–	–

*The complete structures (payload + linker + amino acid) of Belantamab mafodotin and Trastuzumab emtansine have been considered for the calculation of their properties, due to the no-cleavable nature of the linker

This behavior is maintained if we use other calculations that estimate the drug potential of the molecules, such as the Ghose, Veber, Egan or Muegge rules, the prediction of Leadlikeness violations, gastrointestinal (GI) absorption or the Bioavailability Score (Additional file 1: Table S1) [24–30]. Similar findings were observed considering the AB-MPS score, which in all cases exceeds the threshold of 14, but in the cases of Mirvetuximab soravtansine (Elahere), Sacituzumab govitecan (Trodelvy) and Trastuzumab deruxtecan (Enhertu) the values were relatively close to this limit (in the cases where the calculation could be performed). Taking this data into consideration, it can be concluded that some of the selected payloads used for approved ADCs have inappropriate physicochemical characteristics that could limit their activity by reducing the diffusion of the compound through cellular membranes.

### Pharmacokinetic data and schedule of administration

Most of the approved ADCs have used a schedule of administration based on a Q3W regimen. These include belantamab mafodotin (Blenrep), brentuximab vedotin (Adcetris), loncastuximab tesirine (Zynlonta), mirvetuximab soravtansine (Elahere), polatuzumab vedotin (Polivy), tisotumab vedotin (Tivdak), trastuzumab emtansine (Kadcyla) and trastuzumab deruxtecan (Enhertu). ADCs with a more frequent administration, mainly D1, D8 every 21 days or D1, D8 and D15 every 28 days, include enfortumab vedotin (Padcev), gemtuzumab ozogamicin (Mylotarg), inotuzumab ozogamicin (Besponsa) and sacituzumab govitecan (Trodelvy) (Table 3). Enfortumab vedotin (Padvec) and Sacituzumab govitecan (Trodelvy) are administered in solid tumors and gemtuzumab ozogamicin (Mylotarg) and inotuzumab ozogamicin (Besponsa) in hematological malignancies. The more frequent administration of the compound could be, in some cases, due to issues related to target-mediated drug disposition (TMDD) secondary to a high expression of the TAA in normal tissue, so a higher proportion of the antibody is needed to saturate the TAA in normal tissue. As can be seen in Fig. 1, this could be the case for enfortumab vedotin (Padcev) that targets Nectin-4 or sacituzumab govitecan (Trodelvy) for Trop-2. However, in hematological malignancies the high tumor burden could be also the reason, like for gemtuzumab ozogamicin (Mylotarg) and inotuzumab ozogamicin (Besponsa).

**Table 3:** Pharmacokinetic characteristics and schedule of administration of approved ADCs. Information about the FDA-approved medical indication, target and payload is also provided

Drug name	Condition	Target	Payload (MW)	Half-life	Dose	Frequency
Belantamab mafodotin (Bienrep)	MM > 3 lines, including anti-CD38, proteasome inhibitor and one immunomodulator	BCMA	MMAF (732)	ADC: 11.5 days	2.5 mg/kg (Max 125 mg)	Q3W
			cys-mcMMAF (925)			
Brentuximab vedotin (Adcetris)	HL Ill or IV treatment-naive	CD30	MMAE (718)	ADC: 3.79–4.43 days	1.8 mg/kg	Q3W
				Free drug: 3–4 days		
Enfortumab vedotin (Padcev)	Advanced urothelioma that has received anti-PD-(L)l and platinum	Nectin-4	MMAE (718)	ADC: 3.4 days	1.25 mg/kg	Days 1, 8 and 15 every 28 days
				Free drug: 2.4 days		
Gemtuzumab ozogamicin (Mylotarg)	AML CD33 + treatment-naïve Elderly, ECOG 2	CD33	Calicheamicin (1368)	ADC: 160h (6 days)	3 mg/m^2^/dose	Days 1, 4 y 7 induction phase
Inotuzumab ozogamicin (Besponsa)	Relapsed or refractory ALL CD22 +	CD22	Calicheamicin (1368)	ADC: Cycle 1: 6 days Cycle 4: 13 days	First cycle: 1.8 mg/m^2^ Next cycles if complete response: 1.5 mg/m^2^ Next cycles if not complete response: 1.8 mg/m^2^	First cycle: 0.8 mg/m^2^ day 1 0.5 mg/m^2^ day 8 and 15 After the first three weeks: 0.5 mg/m^2^ on days 1, 8 y 15 every 28 days in case of complete response
Loncastuximab tesirine (Zynlonta)	DLBCL or HGBL. > 1 previous line	CD19	SG3199 (585)	ADC: Cycle 1: 14.6 days Cycle 5: 20.8 days	0.15 mg/kg in the first two cycles 0.075 mg/kg from cycle 3 on	Q3W
Mirvetuximab soravtansine (Elahere)	Ovarian cancer. Platinum-resistant. 1–3 previous lines. FRa positive	FRa	DM4 (780)	ADC: 4 days	6 mg/kg	Q3W
				Free drug: 2.6 days		
Polatuzumab vedotin (Polivy)	With rituximab, cyclophosphamide, doxorubicin, and prednisone (R-CHP) for adult patients who have previously untreated diffuse large B-cell lymphoma (DLBCL), not otherwise specified (NOS), or high-grade B-cell lymphoma (HGBL) and who have an InternationaI Prognostic Index (IPI) score of 2 or greater	CD79	MIMAE (718)	ADC: 12 days (cycle 6)	1.8 mg/kg	Q3W
				Free drug: 3.8 days (cycle 1)		
Sacituzumab govitecan (Trodelvy)	mTNBC > 2 previous lines. mLBC. Endocrine therapy, CDK-i and > 2 previous CT	Trop-2	SN-38 (392)	ADC: 16h	10 mg/kg	1 and 8 every 21 days
				Total drug: 18h		
				Free drug: 18h		
				Metabolite: 15h		
Tisotumab vedotin (Tivdak)	Metastatic cervix uterii. 1–2 previous lines	Tissue factor	MMAE (718)	ADC: 4 days	2 mg/kg (Max 200 mg)	Q3W
				Free drug: 2.6 days		
Trastuzumab Deruxtecan (Enhertu)	MBC HER2 + , previous line with a taxane and trastuzumab HER2 + eBC with residual disease after NACT with taxane and trastuzumab mCM HER2 + . Previous taxane and trastuzumab, no T-DMl	HER2	DM1 (734)	ADC: 4 days	3.6 mg/kg	Q3W
Trastuzumab Emtansine (Kadcyla)	mCM HER2-low. HR + and HR-1–2 previous CT	HER2	Dxd (1034)	ADC: 5.4–5.7 days	5.4 mg/kg (breast and Iung) 6.4 mg/kg (gastric)	Q3W
	mNSCLC. HER2 activating mutations > lL CT Gastric o GEJ HER2 + . Previous trastuzumab, FluorP and platinum agent and > 2 L			Free drug: 5.4–6.1 days		

Another interesting observation is the fact that the half-life of the ADC does not cover the schedule of administration. For most ADCs the half-life is around one week, although the administration is given Q3Ws. Remarkable, one ADC, Sacituzumab govitecan, shows a half-life as short as 16 h although the agent is dosed at D1 and D8 every 21 days.

### Clinical efficacy

Up to twelve ADC have been approved by the FDA by 2023, although one of them, belantamab mafodotin (Blenrep), has recently been withdrawn from the US market upon sponsor request to the FDA. Five of them have been developed and approved for the treatment of solid tumors, while the other eight have been granted approval for the treatment of hematological malignancies. However, it must be noted that, while most of the older ADC have phase III clinical trials supporting their use, some of the recent ADC approvals are based on phase II trials (Table 4).

**Table 4:** Characteristics of clinical trials used for the approval of the ADC including design, indication, line of treatment, endpoint and efficacy data. The last column displayed information, when available, about the clinical activity of the previous standard of care in the indication of approval

ADC	Trial	Population	Design	Primary endpoint	Results
Belantamab mafodotin	DREAMM-2. NCT03525678	MM > 3 lines, including anti-CD38, proteasome inhibitor and one immunomodulator	Phase 2. Open label trial of BM monotherapy with two cohorts: BM at 2.5 mg/kg and 3.4 mg/kg	ORR	ORR 31% at 2.5 mg/kg
					DOR > 6 months 73%
Brentuximab vedotin (adults)	ECHELON-1	LHc III or IV treatment-naive	Phase 3, randomized 1:1 A + AVD versus ABVD	Modified PFS	mPFS not reached at approval, with HR 0.77 y p 0.035
	NCTOl712490				
Enfortumab vedotin	EV-301	Advanced urothelioma that has received anti-PD-(L)1 and platinum	Phase 3 randomized 1:1 EV versus TPC	OS	OS 12.88 versus 8.97 m. HR 0.70
	NCT03474107				PFS 5.55 versus 3.71 m. HR 0.62
Gemtuzumab ozogamicin monotherapy (adults)	AML-19	AML CD33 + treatment-naive. Elderly, ECOG 2	Phase 3, randomized, M versus BSC	OS	OS 4.9 versus 3.6 m
	NCT00091234				HR 0.69
Gemtuzumab ozogamicin combination (adults)	ALFA-0701 NCT00927498	AMLCD33 + treatment-naive	Phase 3, randomized 1:1 a DA-M versus DA	EFS	2-y EFS: 17.1% versus 40.8%
					HR 0.58
lnotuzumab ozogamicin	INO-VATE ALL NCT01564784	Relapsed or refractory ALL CD22 +	Phase 3, randomized 1:1 de 10 versus TPC	CR and OS	CR 80.7% versus 29.4%
					DOR 4.6 versus 3.1 m
					PFS 5 versus 1.8 m
					OS 7.7 versus 6.7 m
Loncastuximab tesirine	LOTIS-2. NCT03589469	DLBCL or HGBL. > 1 previous line	Single-arm phase 2 LT monotherapy	ORR	ORR48.3%
					CR 24.1%
					DOR 10.3 months
Polatuzumab vedotin	POLARIX trial. (NCT03274492)	With rituximab, cyclophosphamide, doxorubicin, and prednisone (R-CHP)for adult patients who have previously untreated diffuse large B-cell lymphoma (DLBCL), not otherwise specified (NOS), or high-grade B-cell lymphoma (HGBL) and who have an International Prognostic Index (IPI) score of 2 or greater		PFS	PFS: 76.7% vs. 70.2% at 2 years; HR: 0. 73
Sacituzumab govitecan	TROPiCS-02.NCT03901339	mLBC. Endocrine therapy, CDK-i and > 2 previous CT	SG versus TPC 1:1	PFS	(HR 0.66)
					ORR 21% versus 14%
Sacituzumab govitecan	ASCENT. NCT02574455	mTNBC	Phase 3. Randomized	PFS in the CNS disease-free population	PFS 5.6 versus 1.7 m
		> 2 previous lines	SG versus TPC 1:1		OS 12.1 versus 6.7 m
					ORR 35% versus 5%
Tisotumab vedotin	lnnovaTV 204	Metastatic cervix uterii. 1–2 previous line including platinum agent	Single-arm phase 2. TV monotherapy	ORR and DOR	ORR 24%
	NCT03438396				DOR 8.3 months
Trastuzumab deruxtecan	DESTINY-Breast03	CMm HER2 + . Previous taxane and trastuzumab, no T-DMl	Phase 3, randomized. T-DXd versus T-DMl 1:1	PFS	PFS 28.8 versus 6.8
	NCT03529110				OS NR, HR 0.64
					ORR 79.7 versus 34.2%
Trastuzumab deruxtecan	DESTINY-Breast04	CMm HER2-low. HR + and HR-	Phase 3, randomized. T-DXd versus TPC 2:1	PFS in HR + population	PFS 10.1 versus5.4 m
	NCT03734029	1–2 previous CT			05 23.9 versus 17.5 m
					ORR 52.6% versus 16.3%
Trastuzumab deruxtecan	DESTI NY-GastricOl	Gastric o GEJ HER2 + . Previous trastuzumab, FluorP and platinum agent and > 2 L	Phase 2. Randomized with T-DXd 6.4 mg/Kg versus TPC	ORR	ORR 40.5% versus 11.3%
	NCT03329690			OS key secondary endpoint	OS 12.5 versus 8.4 m
					HR 0.59
Trastuzumab emtansine	EMILIA NCT00829166	MBC HER2 + , previous line with a taxane and trastuzumab	Phase 3, randomized 1:1 de T-DMl versus lapatinib-capecitabine	PFS y 05	PFS 9.6 m versus 6.4 m
					OS 30.9 versus 25.1 m
Trastuzumab emtansine	KATHERINE NCT01772472	HER2+ eBC with residual disease after NACT with taxane and trastuzumab	Phase 3, randomized 1:1 T-DMl versus trastuzumab	iDFS	3-y iDFS 88.3% versus 77%, HR 0.50

Gemtuzumab ozogamicin (Mylotarg), brentuximab vedotin (Adcetris), trastuzumab emtansine (Kadcyla), inotuzumab ozogamicin (Besponsa), enfortumab vedotin (Padcev), trastuzumab deruxtecan (Enhertu), sacituzumab govitecan (Trodelvy) and polatuzumab vedotin (Polivy) have been approved based on phase III studies. Consequently, the endpoints used to demonstrate clinical activity were mainly OS, PFS, EFS and iDFS. In specific indications, the approval of trastuzumab deruxtecan (Enhertu) has been based on data from phase II trials with ORR as their primary endpoint like in gastric and NSCLC [31, 32] Belantamab mafodotin (Blenrep), loncastuximab tesirine (Zynlonta), tisotumab vedotin (Tivdak) and mirvetuximab soravtansine (Elahere) have been approved with data from phase II studies, using ORR and DOR, as primary endpoints. Those accelerated approvals are pending to be confirmed in further phase III registrational studies.

As previously stated, it must be noted that belantamab mafodotin (Blenrep) was granted approval based on the phase II DREAMM-2 trial that had ORR as its primary endpoint. The phase III DREAMM-3 trial (NCT04162210), that compared belantamab versus pomalidomide and dexamethasone, with PFS as its primary endpoint, resulted to be negative. Therefore, on November 22, 2022, the sponsor announced the withdrawal of the compound following the FDA request [33].

On the contrary, polatuzumab vedotin (Polivy) was granted approval in pretreated DLBCL in 2019. Its approval was based on a phase Ib/II trial that had CR rate as its primary endpoint. Recently, this drug has confirmed its activity in a phase III trial in pretreated patients [34], and the FDA has granted full approval in adults who have previously been untreated with diffuse large B-cell lymphoma (DLBCL), not otherwise specified (NOS), or high-grade B-cell lymphoma (HGBL) and who have an International Prognostic Index (IPI) score of 2 or greater[35].

## Discussion

In the present article, we analyze the components present in the structure of ADCs that should be taken into consideration when exploring activity and safety of this family of agents.

We have first recognized that the number of TAAs exploited for approved ADCs is limited, and that the differential expression between tumoral areas and normal tissues is narrow in most solid tumors compared to hematological malignancies. This observation suggests two important implications: The first one is that a huge differential expression between the TAA in tumor and normal tissue, while desirable for safer therapeutic index, is not a mandatory requirement for the development of a specific ADCs. The second implication is that the identification of novel TAAs is necessary to widen the therapeutic spectrum against different cancers. Beyond this work, other recent articles have focused on potential therapeutic opportunities for clinical development of approved ADCs in indications not yet exploited [9].

We observed that most of the approved ADCs used cleavable linkers that release the payload under certain conditions. Among them, most were dependent on enzymatic activity and only two were related to pH conditions. An important observation from those that use a cleavable linker, for the payload to be released, is that they need to be degraded by proteases or by the change of pH within the lysosomes. In this context, changes in the lysosome pH that induces an abnormal protein degradation can affect the diffusion of the payload through the lysosome membrane leading to the development of resistance [10, 36]. Consequently, it would be preferable to develop cleavable linkers that would release a free payload. However, it is unclear which type of cleavable linker would be superior.

Payload characteristics have not been taken into consideration when evaluating the activity of the ADCs. In our analysis, we have evaluated the physicochemical characteristics of ADC-approved payloads identifying that only two of them, DXd and SN-38, qualified for these rules. This finding suggests that some of the payloads will have limitations when diffusing through biological membranes, therefore reducing the amount of compound that will bind to the target. The development of future ADCs should take into consideration the physicochemical characteristics of the payloads beyond the mere evaluation of the mechanism of action and potency. In this context, some recent articles have reported ADCs with optimized payloads with more potent antitumoral activity [37].

The action of an ADC is not exclusively produced by the internalization of the payload in the cell, but also to the subsequent diffusion of the molecule through membranes leading to the induction of a bystander effect or bystander killing [38]. For this reason, physicochemical characteristics of the molecules, such as their solubility, lipophilicity or size (parameters considered in the different Leadlike rules), largely determine the possibility of diffusion and transport from one cell to another through nearby membranes [39].

The bystander effect is the ability of a certain ADC to exert its cytotoxic activity in cells that do not express the target antigen. It requires the payload to cross the targeted-cell membrane to act upon non-target expressing neighboring cells. It requires the payload to be hydrophobic and non-polar. A cleavable linker is also preferred since the linker-payload structure is less likely to be able to cross cell barriers [40, 41]. In a similar manner a payload with adequate physicochemical characteristics can facilitate this process. An adequate example of an ADC with bystander effect is Trastuzumab deruxtecan, that has been approved in indications with low target expression, as shown in Table 4. Other appropriate examples include Sacituzumab govitecan, with SN-38 as a payload.

Future ADCs should be designed to produce a bystander effect targeting indications with overexpression but also mid to low expression of the TAA. To this regard some ADCs are exploring their effect in ultra-low TAA expression tumors, particularly for those with bystander effect [42].

We also evaluated the in vitro potency and the DAR of all the approved ADCs, identifying that these parameters are not a key factor alone for the development of this type of agents. However, it is of note that those payloads compliant with Lipinski rules (and others) pose a limit in the achievable DAR, since hydrophobicity of the payload could promote aggregation and affects stability of the ADC. Indeed, trastuzumab deruxtecan (Enhertu) and sacituzumab govitecan (Trodelvy) harbor the highest DAR (around 8). Another interesting observation is the increase in potency of the ADC compared with the payload for some compounds including sacituzumab govitecan, trastuzumab deruxtecan or loncastuximab tesirine (Table 3). These three agents have a cleavable linker, and sacituzumab govitecan and trastuzumab deruxtecan have a payload with favorable physicochemical characteristics and a high DAR. These data align with recent publications discussing the therapeutic index of ADCs and ways to optimize their administration to improve tolerability [43, 44]

In line with this, another important aspect is the schedule of administration to achieve and maintain target engagement and biological activity. As described in Table 3, two interesting observations can be made. The first one is that a more frequent schedule of administration could be secondary to a TMDD. However, this is not exclusive, as can be seen also for targets highly expressed in the tumor like those in hematologic malignancies. Secondly, the schedule of administration does not match the ADC half-life, which suggests that the biological activity could be optimized with more frequent administrations of the compound. A very nice analysis of the pharmacokinetic properties of approved ADCs suggests that a more frequent administration can increase systemic payload concentrations for some of the ADCs [45]. These two observations provide insights into the best way to develop novel ADCs and reinforce the suggestions promoted by the Optimus project [46]. In this context, exposure–response relationships for efficacy should be optimized aiming to use the minimum biological active dose.

Finally, when exploring the clinical efficacy data, we observed that some compounds were approved based on a non-time to event endpoints using single-arm phase II studies. These approvals were performed following a FDA-accelerated path that requires subsequent confirmation with full registrational phase III studies. Although most of the studies met the endpoints for full registration, some did not and were withdrawn, as described in the result section, for belantamab mafodotin (Blenrep). Of note, the recently released FDA guidelines for accelerated approval suggest that randomized studies with time to event endpoints should be performed if aiming for an accelerated approval [47, 48]. Therefore, future development of this type of agents should be executed in a different manner as it has been done in recent times.

Our study has limitations. For the evaluation of the presence of TAA, we used genomic data and not proteomic data. In addition, this information was obtained from publicly available genomic datasets. Of note, we are not considering in this article antibody characteristics like specificity, affinity, antibody-receptor internalization, or recycling, among others [4, 10, 49, 50]. In addition, the mechanism of action of the payload in relation with the tumor sensibility has not been evaluated, as no data to perform such analysis exist (since the MoA for currently approved ADCs is typically unspecific). ADCs using targeted small molecules are currently in early clinical development but are not the scope of this article.

Taking into consideration all the data provided here we could suggest that the best-case scenario for the development of an ADCs should match the following characteristics: (1) the selection of a specific TAA only expressed in tumoral tissue, (2) the use of a cleavable linker and (3) the use of a payload with adequate physicochemical characteristics. Our suggestion for the best-case scenario is a payload with good physicochemical characteristics, in an ADC with a moderate to high DAR, independently of the in vitro potency of the payload, like is the case for sacituzumab govitecan. From a clinical point of view consideration should be given for novel FDA guidelines for dose optimization and an accelerated approval path [48]. In line with this, optimization of the schedule of administration using a more frequent one could improve the therapeutic index leading to the presence of higher amounts of free payload in the systemic circulation.

In summary, by evaluating currently approved ADCs, we provide novel ideas to be considered for the design of next-generation ADCs for cancer.

### Supplementary Information

**Additional file 1**. **Table S1.** Frequency of breaches in the parameters of distinct rules pertaining to the pharmacological potential of molecules. Furthermore, assessment of gastrointestinal (GI) absorption and bioavailability score, both of which serve as metrics for evaluating the permeability of compounds across the intestinal barrier. **Table S2.** Parameters included in the different rules, in addition to the definition of gastrointestinal absorption parameters and Bioavailability Score.

## Data Availability

All data generated or analyzed during this study are included in this published article.
