# Supplementary material for: Considerations for the design of antibody drug conjugates (ADCs) for clinical development: lessons learned
Source: J Hematol Oncol. 2023 Dec 12;16:118. doi: 10.1186/s13045-023-01519-0 (PMC10717055; doi:10.1186/s13045-023-01519-0)
Supplement: Supplementary file 1 — Additional file 1. Table S1. Frequency of breaches in the parameters of distinct rules pertaining to the pharmacological potential of molecules. Furthermore, assessment of gastrointestinal (GI) absorption and bioavailability score, both of which serve as metrics for evaluating the permeability of compounds across the intestinal barrier. Table S2. Parameters included in the different rules, in addition to the definition of gastrointestinal absorption parameters and Bioavailability Score. [file 13045_2023_1519_MOESM1_ESM.docx]

**Supplemental information**

**Data extraction**

In order to identify FDA-approved ADC, we researched the FDA website (last access July 2023). The FDA has a publicly available database termed: “Novel Drug Approvals” that is updated every year. We thoroughly examined the available list to identify drugs that are considered ADC and have been approved.

**Preclinical and clinical data criteria**

The preclinical data collected for this analysis is limited to the *in vitro* activity in cytotoxicity assays as reported by the sponsors and extracted from the FDA label. The IC50 of the free drug is compared to the IC50 of the ADC. A data range is generally provided by the sponsors, the lower one representing the activity on sensitive cell lines, and the higher one for non-sensitive cell lines.

The clinical data collected for this analysis is related to the pharmacokinetic of the ADC plus that of the payload and metabolites (if appropriate). Specifically, the Cmax and the half-life parameters have been extracted from the corresponding labels cited. Some products have several labels available in the FDA site, as a result of label revisions, which may or may not contain the exact same data (since the sponsor may choose a different clinical study to document Cmax and half-life in a revised label), as in the label cited in this work.

**Transcriptomic extraction and data analysis**

Using public available datasets (Gene Expression Profiling Interactive Analysis; <http://gepia2.cancer-pku.cn/> using TCGA)(1) we analyzed the expression (in transcripts per million, TPM) of these targets in the tumoral and normal tissue of all cancer types. We selected those targets whose TPM was equal to or greater than 32, a value considered medium/high in terms of gene expression.

We also explored the genes that are dependent in cell lines (<https://depmap.org/portal/>).

**Evaluation of physicochemical characteristics of the payload and definitions**

Among all the physicochemical parameters (e.g. lipophilicity, octanol/water partition coefficient or logP, solubility, topological polar surface area...), pharmacokinetic and possible drug-likeness rules, the rule of 5 or Lipinski rule was selected for evaluation. The choice of this rule was made on the basis that it is a frequently used concept in the field of Drug Discovery, and that it is related to the permeability of a drug comprising several individual parameters. The Lipinski rule predicts that a drug will have good pharmacokinetic properties (absorption, distribution, metabolism and excretion based on the following specific physicochemical properties: *(1)* No more than 5 hydrogen bond donors, *(2)* No more than 10 hydrogen bond acceptors, *(3)* Molecular weight less than 500 Da, and *(4)* LogP not greater than 5. Also, and beyond the Rule of 5, there are other parameters that are related with good oral absorption, like the AB-MPS score. This parameter is often related with acceptable oral absorption of compounds, with AB-MPS values ≤ 14 predicting a higher probability of success. Unlike stricter pass/fail metrics that rely on cutoff values, AB-MPS provides a continuum of scores to evaluate drug-likeness (2).

All ADME parameters (Absorption, Distribution, Metabolism, and Excretion), including Lipinski rule, were calculated using SwissADME, a free software program available from the Swiss Institute of Bioinformatics. In addition, each of the parameters that constitute the Lipinski rule were broken down (3). Also, the average calculation of the LogP and LogS parameters obtained by different algorithms in the software was included, to highlight the lipophilicity and solubility of each payload. cLogD values were obtained from the ChEMBL database (when available) (4), and AB-MPS score was calculated using the following formula: AB-MPS = Abs(cLogD - 3) + N_AR_ + N_RB_ were Abs means absolute value, N_AR_ is the number of aromatic heavy atoms, and N_RB_ is the number of rotatable bonds in the molecule. Additional parameters (Additional file 1 and 2) were also evaluated and described.

Additional file 1: Supplementary Table 1. Frequency of breaches in the parameters of distinct rules pertaining to the pharmacological potential of molecules. Furthermore, assessment of gastrointestinal (GI) absorption and bioavailability score, both of which serve as metrics for evaluating the permeability of compounds across the intestinal barrier.


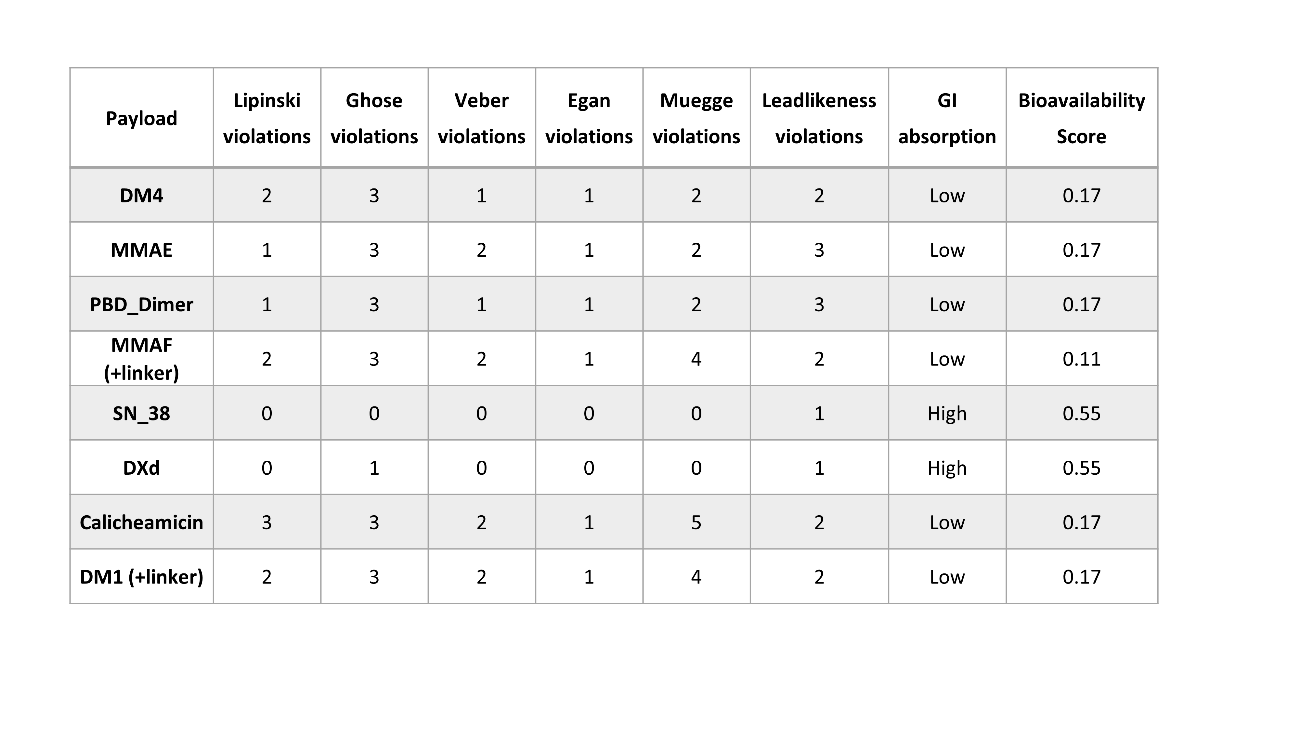


Additional file 1: Supplementary Table 2. Parameters included in the different rules, in addition to the definition of gastrointestinal absorption parameters and Bioavailability Score.


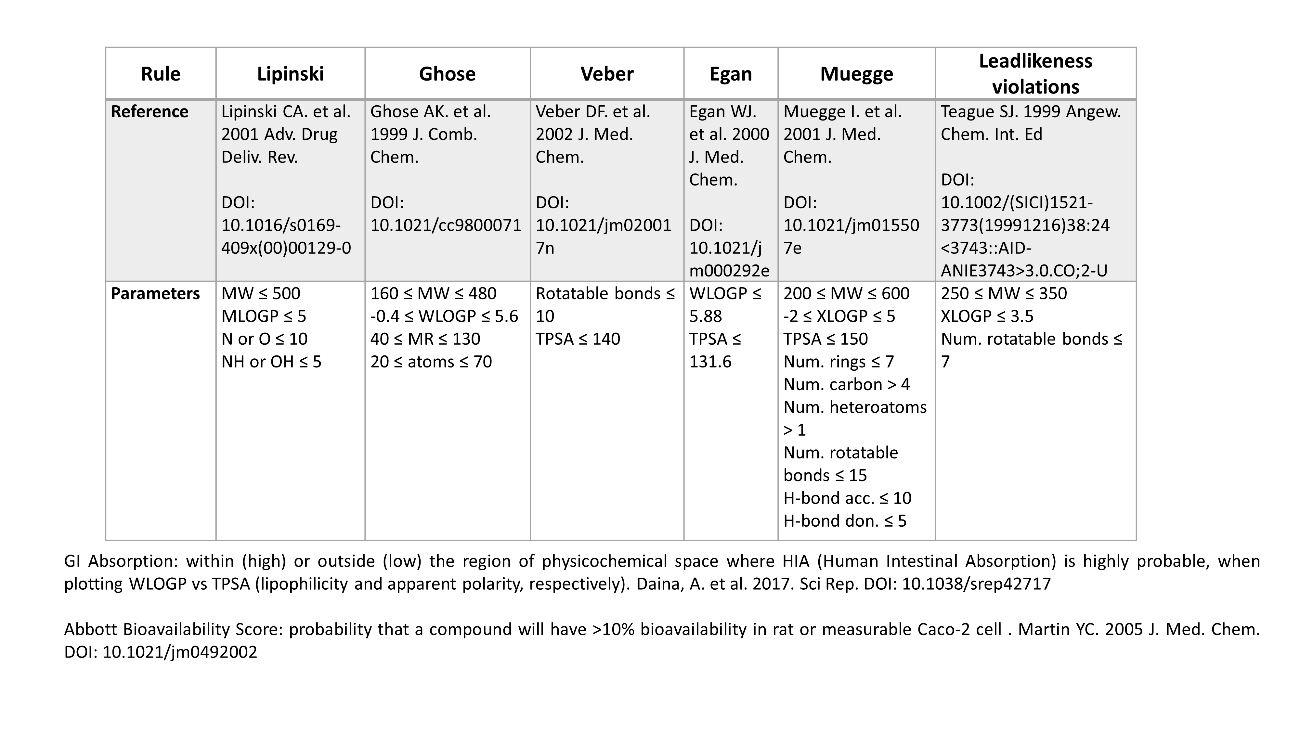


1. Tang Z, Kang B, Li C, Chen T, Zhang Z. GEPIA2: an enhanced web server for large-scale expression profiling and interactive analysis. Nucleic Acids Res [Internet]. 2019 Jul 1 [cited 2023 Aug 22];47(W1):W556–60. Available from: https://pubmed.ncbi.nlm.nih.gov/31114875/

2. Degoey DA, Chen HJ, Cox PB, Wendt MD. Beyond the Rule of 5: Lessons Learned from AbbVie’s Drugs and Compound Collection. J Med Chem [Internet]. 2018 Apr 12 [cited 2023 Nov 23];61(7):2636–51. Available from: https://pubs.acs.org/doi/abs/10.1021/acs.jmedchem.7b00717

3. Daina A, Michielin O, Zoete V. SwissADME: a free web tool to evaluate pharmacokinetics, drug-likeness and medicinal chemistry friendliness of small molecules. Sci Rep [Internet]. 2017 Mar 3 [cited 2023 Jul 31];7. Available from: https://pubmed.ncbi.nlm.nih.gov/28256516/

4. ChEMBL Database [Internet]. [cited 2023 Nov 23]. Available from: https://www.ebi.ac.uk/chembl/
